# How Characteristics of Florida’s Assisted Living Communities Impact Services and Recreational Activities Offered

**DOI:** 10.1093/geroni/igaa057.298

**Published:** 2020-12-16

**Authors:** Jocelyn Brown, Daniel Pupo

**Affiliations:** University of South Florida, Tampa, Florida, United States

## Abstract

There are currently 3,090 assisted living communities (ALCs) serving older adults throughout the state of Florida. The services (e.g. physical therapy) and recreational activities (e.g. cooking classes) offered within these communities likely differ depending on a variety of characteristics such as location, licensure type, and profit status. The goal of this work is to determine how these characteristics influence the number and types of services and activities are offered within Florida’s ALCs. Data on the services and recreational activities, location, and characteristics of ALCs were collected from the state Agency for Health Care Administration (AHCA) website. Counties were classified as rural or urban and based on data from the 2010 U.S. Census. Linear regressions were used to model the associations. The results indicated that rural-based ALCs provided significantly fewer services and activities, compared to urban-based ALCs. ALCs that were for-profit, with more licensed beds and with a limited nursing service license showed increasing numbers of services and activities, while ALCs with limited mental health licenses showed significant decreases. ALCs that are rural, non-profit or that hold limited mental health licenses to provide fewer services and recreational activities for residents than ALCs without these characteristics. Policymakers and administrators should work to ensure that residents living within these communities have adequate access to services and activities by addressing administrative, logistical, and financial barriers.

